# Bonding the Titanium Attachment Matrix Housing to Acrylic Overdentures Using Different Primers, Adhesives, and Resin Materials: An In Vitro Study

**DOI:** 10.1155/ijod/9702318

**Published:** 2025-04-08

**Authors:** Mohammadreza Nakhaei, Hosein Dashti, Sepideh Sasannejad, Hamideh Sadat Mohammadipour

**Affiliations:** ^1^Dental Materials Research Center, Department of Prosthodontics, School of Dentistry, Mashhad University of Medical Sciences, Mashhad, Iran; ^2^Department of Prosthodontics, School of Dentistry, Mashhad University of Medical Sciences, Mashhad, Iran; ^3^Zahedan University of Medical Sciences, Zahedan, Iran; ^4^Dental Materials Research Center, Department of Restorative Dentistry, School of Dentistry, Mashhad University of Medical Sciences, Mashhad, Iran

**Keywords:** alloy primer, attachment matrix housing, bond strength, housing attachment, implant attachment housing, overdenture, Ti housing, universal adhesive

## Abstract

**Background and objectives:** Dislodgement of attachment matrix housing (AMH) from acrylic resin base is considered a major problem in overdentures. It is necessary to achieve a strong and durable bond between AMH and the acrylic part. The application of primers, adhesives, and resin materials containing multifunctional monomers may improve their adhesion. The purpose of this in vitro study was to evaluate, the effects of bonding protocols on shear bond strength (SBS) of resin materials to Ti and denture acrylic base.

**Methods:** Sixty poly methyl methacrylate (PMMA) blocks (15 × 15 × 15 mm) and 60 AMH were prepared and divided into six groups (*n* = 10). The acrylic resin specimens were bonded using one of the following adhesive strategies: (P) flowable resin composite, (A-SR-P) air abrasion + SR Connect primer + flowable resin composite, (A-SR-Q) air abrasion + SR connect primer + Quick up, (A-SR-MHA) air abrasion + SR Connect primer + autopolymerizing luting composite, (Q) Quick up and (T) autopolymerizing acrylic resin. The surfaces of the Ti housing were bonded using one of these protocols: (GP-P): G-Premio BOND + light-cured resin composite, (AP-GP-P) ALLOY PRIMER + G-Premio BOND + resin composite, (Q) Quick Up, (AP-Q) ALLOY PRIMER + Quick Up, (MP-MHA) Monobond Plus primer + Multilink Hybrid Abutment, and (T) autopolymerizing acrylic resin. After thermal cycling (5000 cycles), the SBS was evaluated and the failure modes were recorded. Data (MPa) were analyzed using Shapiro–Wilk, one-way ANOVA, the post hoc Tukey HSD, and Games–Howell tests (*α* = 0.05).

**Results:** In the acrylic resin groups, the greatest SBS values were reported in Q (11.97 ± 2.65 MPa), T (15.11 ± 5.36 MPa), and A-SR-P (8.32 ± 4.51 MPa) groups with no significant differences among them (*p*  > 0.05). The SBS of A-SR-MHA and P groups was significantly lower than other study groups. In AMH, the SBS of GP-P (9.36 ± 1.57 MPa), AP-GP-P (7.66 ± 3.35 MPa), and MP-MHA (6.37 ± 2.39 MPa) were noticeably higher than other groups. There was no significant difference in adhesion to Ti between GP-P and AP-GP-P (*p*=0.63), as well as Q and AP-Q (*p*=0.65).

**Conclusions:** For improving the adhesion of AMH to acrylic resin of overdentures, the application of G-Premio BOND with or without ALLOY PRIMER in Ti substrate and Quick Up and autopolymerizing acrylic resin to the acrylic resin of denture base produced the best adhesion. ALLOY PRIMER could not increase the adhesion to Ti substrate.

## 1. Introduction

In recent years, the conventional dentures in edentulous patients have been replaced by the implant-supported overdentures, which improve prosthetic retention and stability, masticatory ability, and quality of life of patients [1]. The implant-supported overdentures are retained by the attachment matrix housing (AMH) system, which is located inside the denture and connected to the implants through either solitary-type or bar-type attachments [2]. The AMH can be inserted into the overdenture structure either through chairside procedure (direct technique) or in a laboratory place (indirect technique). Most practitioners prefer the chairside technique to minimize processing errors [3]. The heat or autopolymerized poly methyl methacrylate (PMMA), light-cured resin composite, relining resins or autopolymerizing resin-based materials are used for pickup of these attachments [1, 4]. PMMA was commonly used for picking up attachments, which showed the highest adhesion between the metal cap and denture base resin [1]. However, due to several drawbacks for both practitioners and patients, including a long setting time, difficult removal of excess material, unfavorable odor and taste, as well as its exothermic reaction, which can burn the patient's mucosa, PMMA has been replaced by other pick-up materials. These materials are relatively tasteless and possess a different chemistry than PMMA, which allows for easier clean-up and a predictable setting time by light-curing processes [4].

The AMH must be strongly bonded to acrylic resin materials to avoid detachment in the humid oral environment. The retention of resin materials to AMH mainly comes from the macromechanical retention through engaging the existing undercuts on the axial walls of the attachment, and no chemical bond formed. Therefore, they are susceptible to debonding from the denture base over time. Reinsertion of the AMH into the denture base is costly and time-consuming, and the masticatory function and esthetic of the patient may be compromised for a short time [1].

Since chemical adhesion is necessary for the success of bonding between the pick-up material with AMH and PMMA denture base [5], numerous investigations have been carried out to discover the best bonding strategies. The airborne particle abrasion of titanium (Ti) and PMMA with aluminum oxide particles, followed by conditioning with silane primer or MMA-based liquid (SR Connect, Ivoclar Vivadent Inc., Liechtenstein) enhanced the adhesion of Ti to the acrylic resin and PMMA [6]. The bonding between resin-based materials and Ti has also been improved using carboxylic or phosphoric acid derivatives [7]. For instance, the application of metal primers (ALLOY PRIMER) improved the adhesion of resin materials to Ti [8] or other metals [9], which is not negatively affected by thermocycling [5]. Furthermore, the multimode or universal adhesives, which consisted of functional monomers of 10-MDP and 4-META can adhere the resin materials to different substrates such as base metal, noble metals, Ti, and PMMA [10, 11]. A previous study revealed that the application of metal primers before a multimode adhesive can improve the adhesion of resin cement to the Ti surface, which endures limited thermal aging (5000 cycles of thermocycling) [8].

Based on the aforementioned statements, the selection of the materials and techniques is crucial for producing the greatest and durable adhesion between AMH and acrylic resin when inserting implant attachments into the denture base. While previous research has explored the bond strength of various retaining materials and surface treatments (including airborne particle abrasion), on Ti and acrylic resin (1, 2, 4–6), it remains unclear which of these methods offers the most effective bonding strategy to resist dislodgement forces. Therefore, this study aims to compare the shear bond strength (SBS) of autopolymerizing acrylic resin (SR Triplex Cold Pink), light-cured resin composite (PermaFlo Pink), autopolymerizing resin composite (Quick UP), and resin cement (Multilink Hybrid Abutment) for direct transfer of AMH to heat-polymerized PMMA (SR Triplex Hot Pink) using different primers (ALLOY PRIMER, SR Connect, Monobond Plus) and multimode adhesive (G-Premio BOND) containing functional monomers. So, two null hypotheses were considered in this investigation: (1) the SBS of the acrylic resin and AMH would not be affected by the application of different mentioned resin materials, primers and adhesive, (2) the utilization of ALLOY PRIMER would not be able to improve the SBS of retaining materials to Ti substrate.

## 2. Materials and Methods

### 2.1. Study Design

This study was conducted at the Department of Prosthodontics (Mashhad University of Medical Sciences, Iran). The local ethical committee of Mashhad University of Medical Sciences, Iran, independently reviewed and approved this in vitro study with the protocol number IR.MUMS.DENTISTRY.REC.1398.043.

### 2.2. Sample Size Calculation

A pilot study was conducted and the primary mean and standard deviation of the study groups were recorded to determine the sample size. Based on data of the pilot study using an alpha of 0.01 (type I error probability), and beta of 0.2 (type II error) and power of 80%, the sample size was calculated to be seven samples for each group to be able to reject the null hypotheses. Considering the potential dropout of samples during preparation, the number of samples was increased to 10 samples in each group. Sample size calculation was done using G^*∗*^ Power, version 3.1.9.6 for MS Windows (Franz Faul, Universität Kiel, Germany).

### 2.3. Acrylic Resin Preparation

A total of 60 heat-polymerized PMMA cubes (SR Triplex Hot Pink, Ivoclar Vivadent, Schaan, Liechtenstein) with 15 mm × 15 mm × 15 mm dimensions were prepared. The PMMA blocks were prepared by investing metal patterns in a conventional denture flask. Putty impression material (Pala Lab Putty, Heraeus Kulzer, GmbH) was placed around the metal patterns to facilitate the removal of the processed PMMA from the flask. The heat-polymerized denture base resin (Triplex Hot, Ivoclar Vivadent, Schaan, Liechtenstein) was mixed at a powder/liquid ratio of 2.34 g/mL and packed into the flask following the manufacturer's instructions. Then, they were processed in a hot water bath at a temperature of 100°C for 45 min. The flasks were cooled at room temperature for 30 min, and in cold water for 15 min and then the putty impression materials were gently removed. After the immersion of specimens at 37°C water for 24 h, the excess material was removed with a tungsten carbide bur (Meisinger, Neuss, Germany) at 15,000 rpm, and the surfaces were finished with 200- and 600-grit abrasive paper (Starcke, Hoffman Co, Germany). Then, they were cleaned with 70% ethanol and divided into six groups (*N* = 10) based on the following bonding protocols:

*Group 1* (*P*): The flowable resin composite (PermaFlo Pink, Ultradent, South Jordan, USA) was used in this group. The plastic tubes with 5 mm height and 3.8 mm diameter were filled with the resin composite and were then light-polymerized for 20 s with a light curing device (Blue phase 8, Ivoclar Vivadent, Schaan, Liechtenstein) with a minimum power density of 650 Mw/cm^2^. The power density of the light-cured device was checked at first and after every five exposures.

*Group 2* (*A-SR-P*): The resin surfaces were air abraded by 110 µm aluminum oxide particles (Bego, Bremen, Germany) at 0.4 MPa or 2.5 bar pressure for 10 s at 10 mm distance according to the manufacturer's instructions. Next, the abraded samples were thoroughly rinsed and then cleansed with an ethanol solution. Then, the specimens were treated with a light-cured MMA-based conditioner (SR Connect, Ivoclar Vivadent Inc, Liechtenstein) for 30 s, and gently air-dried based on the manufacturer's recommendation. The conditioner was light-polymerized for 40 s and was then bonded with a flowable resin composite (PermaFlo Pink, Ultradent, South Jordan, USA).

*Group 3* (*A-SR-Q*): The samples of this group underwent air abrasion and conditioning using SR connect, similarly to group 2. However, after the conditioner was polymerized, an autopolymerizing resin composite retaining material (Quick Up, Voco, GmbH, Germany) was applied and let polymerize for 3.5 min.

*Group 4* (*A-SR-MHA*): In this group, the same bonding protocol utilized by the two earlier groups, which involved air abrasion and treatment with SR Connect, was followed. Subsequently, an autopolymerizing luting resin composite (Multilink Hybrid Abutment, Ivoclar Vivadent, Schaan, Liechtenstein) was inserted into the plastic tubes and polymerization was performed for 7 min. During this time, the surface of tubes was covered by a glycerin gel (Ivoclar Vivadent, Schaan, Liechtenstein) to prohibit the formation of the air-inhibited layer.

*Group 5* (*Q*): In this group, the Quick Up adhesive (Voco, GmbH, Germany) was used and let dry for 30 s in the air according to the manufacturer's instructions. Then the plastic molds were filled with Quick Up resin material and waited for 3.5 min to be polymerized.

Group 6 (*T*): No surface treatment was used. The polymer and monomer of an autopolymerizing acrylic resin (SR Triplex Cold Pink, Ivoclar Vivadent, Schaan, Liechtenstein) were thoroughly mixed with a ratio of 13 g polymer (powder) and 10 mL monomer (liquid) with a spatula. Then the plastic tubes were filled with the mixture and waited for 15 min to be polymerized.

### 2.4. AMH Preparations

**Figure 1:**
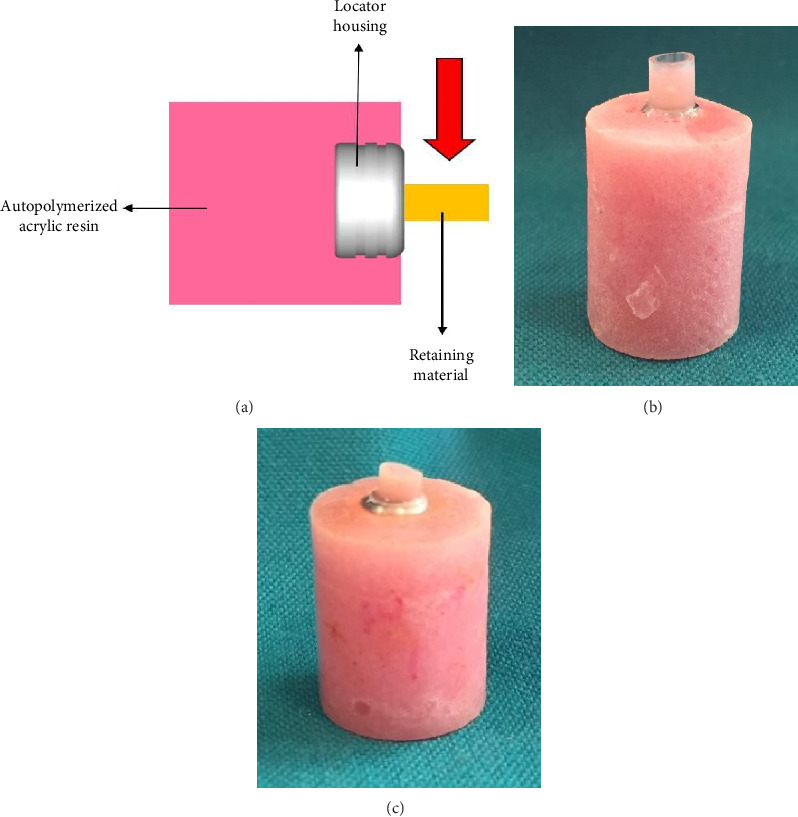
The attachment matrix housing (AMH) with the retaining material which was mounted in self-cured acrylic resin, (a) the schematic view, (b) the plastic mold on the AMH which was filled with the retaining material, and (c) the plastic mold was removed and the retaining material was ready for thermal aging.

The 60 attachment Ti housing matrices (Zest, California, USA) with 5.4 mm diameter and 2.3 mm height were mounted in self-cured acrylic resin cylinders (Acropars, Marlic Co., Tehran, Iran) with 12 mm diameter and 16 mm height in which the flat surfaces of the attachments were above the acrylic resin surface and parallel to the horizon (Figure 1a). Before the surface treatments and bonding, the Ti surfaces were cleaned with 70% ethanol and dried. Then, they were divided into six groups (*N* = 10) based on the surface treatments:

*Group 1* (*GP-P*): The universal or multimode adhesive of G-Premio BOND (GC, Tokyo, Japan) was applied on the surface, and after evaporating the solvent with vigorous air pressure, light-polymerized for 10 s with a light curing device (Blue phase 8, Ivoclar Vivadent, Schaan, Liechtenstein). A flowable resin composite (PermaFlo Pink, Ultradent, South Jordan, USA) was inserted into the plastic molds with 3.8 mm diameter and 5 mm height and polymerized for 20 s (Figure 1b).

*Group 2* (*AP-GP-P*): In this group, before the universal adhesive application, a metal primer (ALLOY PRIMER, Noritake Dental Inc, Kurashiki, Okayama, Japan) (AP) was used for 10 s. Then G-Premio BOND adhesive and resin composite were used according to the group 1 (GP-P).

*Group 3* (*Q*): The adhesive of Quick Up (Quick Up, Voco, GmbH, Germany) was applied on Ti surfaces and dried for 30 s in the air. Then, the plastic mold was filled with Quick Up resin composite retaining material (Quick Up, Voco, GmbH, Germany) and waited for 3.5 min to be polymerized.

*Group 4* (*AP-Q*): Prior to applying the Quick Up adhesive and resin material (Quick Up, Voco, GmbH, Germany), AP (Noritake Dental Inc, Kurashiki, Okayama, Japan) was utilized for a duration of 10 s.

*Group 5* (*MP-MHA*): The attachment surfaces were conditioned with Monobond Plus (Ivoclar Vivadent, Schaan, Liechtenstein) for 60 s. Following this, an autopolymerizing luting resin composite (Multilink Hybrid Abutment, Ivoclar Vivadent, Schaan, Liechtenstein) was inserted into the plastic tubes and allowed to polymerize for 7 min. Throughout this period, the surface of tubes was covered by a glycerin gel (Ivoclar Vivadent, Schaan, Liechtenstein) to prohibit the development of the air-inhibited layer.

*Group 6* (*T*): No treatment was used in this group. The plastic tubes were filled with a mixture of the autopolymerizing acrylic resin (SR Triplex Cold Pink, Ivoclar Vivadent, Schaan, Liechtenstein) and allowed to polymerize for 15 min.

All procedures were carried out by an expert operator. The bonded specimens were stored in distilled water for 24 h in the incubator (Fine Tech, Shin Saeng, Gyeonggi-do, South Korea) at 37°C and 100% humidity to simulate the oral temperature and complete polymerization. After 24 h, the tubes were carefully removed by a surgical blade (SMI, Steinerberg 8, Belgium) (Figure 1c). The prepared samples were subjected to thermal aging by an automated thermal cycling machine (NemoCo., Mashhad. Iran) with water temperatures between 5 and 55°C for 5000 cycles and a 20-s dwell time. The materials that were used in this study and their properties are demonstrated in Table 1.

### 2.5. SBS Evaluation and Failure Mode Assessment

**Figure 2:**
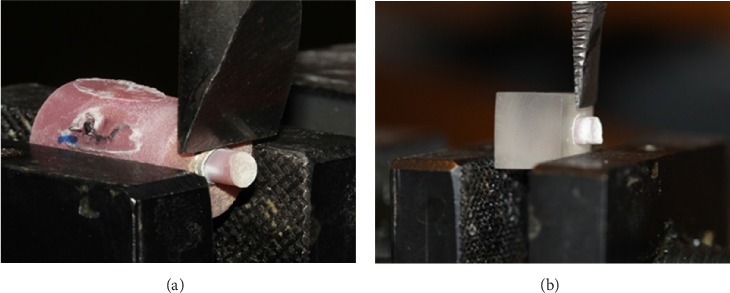
The Ti attachment (a) and acrylic resin specimens (b) were mounted in the universal testing machine for inserting the shear load by a knife-edge chisel to fracture.

The SBS test was performed through inserting the shear load by a knife-edge chisel on a universal testing machine (Santam, model STM-20, Tehran, Iran) at a crosshead speed of 1 mm/min (Figure 2). After the specimens were fractured and removed from the testing apparatus, the fracture sites were observed with the stereomicroscope (Dino lite Pro, Anmo Electronics Corp, Taiwan) at ×30 magnification to identify the type of bond failure. The fracture modes were classified into: (1) adhesive failure at the interface of the AMH or acrylic resin with the bonded materials; (2) cohesive failure within the bonded resin materials (or higher than 50% of resin materials remained on the surface); and (3) the mixed failure mode, a combination of adhesive and cohesive failures (lower than 50% of resin materials remained on the surface).

### 2.6. Statistical Analysis

Data were analyzed using the SPSS statistical software (version 22.0, SPSS Inc., IBM Corp., Armonk, New York, USA). The Shapiro–Wilk test was run to assess the normal distribution of data. One-way ANOVA was used for comparing the SBS of the different study groups. When the differences between groups were statistically significant, the Tukey HSD and Games–Howell post Hoc tests were performed. All the statistical analyses were performed with the significance level set at 5%.

## 3. Results

### 3.1. Acrylic Resin Surfaces

The normal distribution of data was confirmed by Shapiro–Wilk analysis (*p* > 0.05). The descriptive data from acrylic resin bonded specimens, including mean, standard deviation, minimum, and maximum values, were presented in Table 2. The lowest and greatest SBS values were recorded in A-SR-MHA (0.74 ± 0.45) and T (15.11 ± 5.36) groups, respectively. Based on the Levene's test, the homogeneity of variance was confirmed (*p*=0.412). The one-way ANOVA revealed significant differences between study groups (*p* < 0.001). Based on the Tukey test, the mean value of the SBS of the P group was significantly lower than A-SR-Q (*p*=0.029), A-SR-P (*p*=0.024), Q (*p* < 0.001), and T (*p* < 0.001) and significantly higher than the A-SR-MHA group (*p*=0.042). There was no significant difference between the Q, T, and A-SR-P groups (*p*  > 0.05). The SBS values of the A-SR-MHA and P groups were significantly lower than other groups.

### 3.2. AMH

Based on Shapiro–Wilk analysis, the data from AMH groups showed normal distribution (*p*  > 0.05). Mean, standard deviations, minimum, and maximum values of SBS resin materials to AMH surface are shown in Table 3. The lowest and highest mean value of SBS was recorded in Q (2.26 ± 1.98) and GP-P (9.36 ± 1.57) groups, respectively. The one-way ANOVA revealed significant differences between study groups (*p* < 0.001). The SBS of the GP-P group was significantly larger than other study groups except for AP-GP-P (*p* =0.63) and MP-MHA (*p* =0.08) groups. There was no significant difference between T with MP-MHA (*p*=0.52) and Q (*p* =0.8) groups. The mean SBS values of GP-P with AP-GP-P and those of Q with AP-Q did not show significant differences (*p*=0.63 and *p*=0.65, respectively).

**Figure 3:**
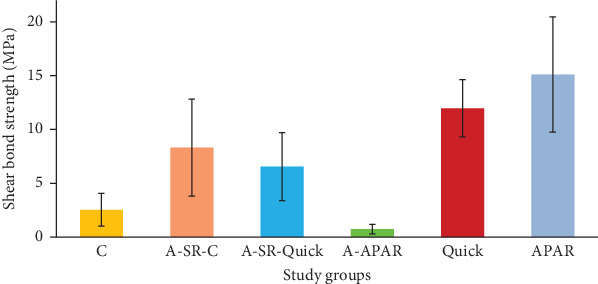
Mean and standard deviation of shear bond strength (SBS) of the study groups in acrylic resin substrate.

**Figure 4:**
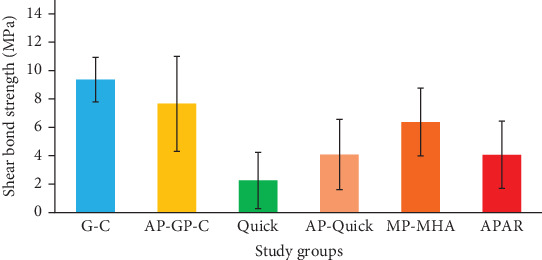
Mean and standard deviation of shear bond strength (SBS) of the study groups in attachment matrix housing (AMH) substrate.

The results of comparison between bonded specimens to AMH and acrylic resin surfaces were demonstrated in Figures 3 and 4, respectively.

### 3.3. Failure Modes

**Figure 5:**
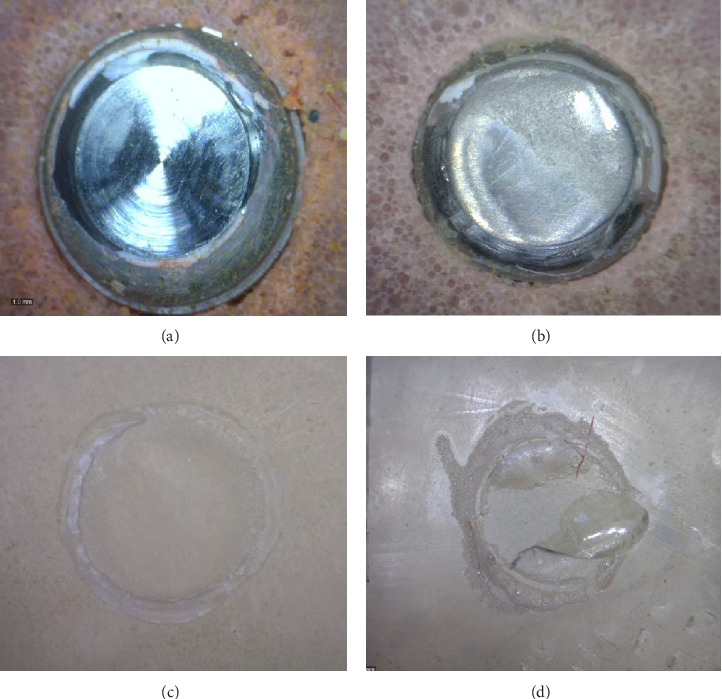
The fracture sites which were observed by the stereomicroscope represented the adhesive and mixed failure modes in (AMH) attachment matrix housing (a and b) and the adhesive and cohesive failure modes in acrylic resin surfaces (c and d).

The modes of failure of all specimens are presented in Table 4. The adhesive failure was reported as the primary failure in all groups (Figure 5a,c), while a few cohesive and mixed failures were seen in the T group in AMH (50% adhesive and 50% mixed failures) and T in acrylic resin substrates (30% adhesive and 70% cohesive failures) (Figure 4b,d).

## 4. Discussion

According to the results of this study, the greatest SBS in acrylic resin substrates was observed in T, Q, and A-SR-P groups and the highest SBS to AMH surfaces was achieved in the GP-P, AP-GP-P, and MP-MHA groups. Since the type of bonding protocol affected the adhesion of the resin materials on acrylic resin and Ti groups, the first null hypothesis of the current research was rejected.

In the present research, the highest SBS values on PMMA specimens were seen in T and Q groups, in which an autopolymerizing acrylic resin and resin composites were used, respectively. In contrast, the group in which the light-cured resin composite was applied revealed the SBS that was significantly lower than other groups, except A-SR-MHA. It was in line with an earlier study by Hirano et al. [12], who revealed that the bonding of autopolymerized resin to the heat-polymerized resin was firmer than light-polymerized resin under both dry and wet conditions. Domingo et al. [6] also reported the same results and showed that the flexural strength (3-point bending test) of autopolymerized acrylic resin to heat-cured acrylic resin was stronger than light-polymerized materials.

The higher SBS values of autopolymerized resins after thermocycling may be explained by the fact that the higher temperature during thermal cycles, used in this study for the aging of the whole samples, enhanced the degree of polymerization of these resins [12]. The low SBS value of the flowable resin composite samples in this study (2.52 ± 1.52 MPa) determined the importance of the selection of resin materials, with the same polymerization mechanisms for repairing denture base or attaching metal parts to PMMA resins. The lower adhesion of the light-cured resin composite to PMMA may be explained by a higher degree of conversion of those materials with no adequate free radicals for bonding to PMMA. In addition, the degree of polymerization of light-cured resins depends on the blue spectrum of visible light and it seems the high temperature has little or no effect on the polymerization of these resins.

The PMMA surfaces of three groups in this study (A-SR-P, A-SR-Q, and A-SR-MHA) were air abraded with 110 micron-sized aluminum oxide particles to remove the outer dense layer on PMMA, increase the surface bonding area, hence, facilitating the penetration of the MMA-based liquid deeper into the surface and increasing the softening of the PMMA with the monomer. However, this method was not effective in the A-SR-MHA group presented the lowest SBS values (0.74 ± 0.45) among the study groups.

Multilink Hybrid Abutment cement is a dimethacrylate and HEMA-based adhesive cement system that requires the use of a primer such as SR Connect and is suitable for the fabrication of temporary abutment crowns made of PMMA, such as Telio CAD. Although the surface conditioning of the PMMA material with MMA-based liquid (SR Connect) was recommended by the manufacturer, it was inadequate to obtain high bonding to the PMMA substrate through autopolymerizing resin materials (Quick Up and Multilink Hybrid Abutment). The greater SBS values in the group where MMA-based liquid treatment was followed by the flowable resin composite (A-SR-P) may be explained by the good copolymerization of light-cured monomers of both the primer and composite. The low retentive strength of PMMA crowns treated by MMA-based liquid and then cemented by Multilink Hybrid Abutment, which was reported by Pitta et al. [11], was in agreement with the present study results. The authors attributed this outcome to two factors. One is related to the high density, high degree of conversion, and few free radicals of PMMA blocks which limited the copolymerization with other resin materials. Indeed, the highly cross-linked surface of PMMA impeded the penetration of MMA-based liquid monomers and then reduced the secondary interpenetrating polymer network (IPN) bonding. The second may be related to the limited time of the application of SR Connect (about 30 s), which is suggested by the manufacturer. It seems this time is too short to wet the PMMA surface and insufficient to initiate the swelling phenomenon that is crucial for secondary IPN bonding [13].

Quick Up is a chairside pickup of attachments with no taste and odor, cures with a low heat through the autopolymerization mechanism, prevents tissue irritation, and provides high bond strength to acrylic resin-based materials. In acrylic resin groups, the Q group in which the autopolymerizing adhesive and resin of Quick Up was used, the SBS value (11.97 ± 2.65) was significantly greater than P, A-SR-Q, and A-SR-MHA groups. This finding was confirmed by previous studies and revealed that the bonding of Quick Up to PMMA denture base resin fulfills clinical requirements [1, 2, 4]. However, in comparison with other pick-up materials such as Jet Denture Repair Acrylic, EZ Pick Up, and UBAR, Quick up revealed the lowest SBS values in Cayouette et al.'s [4] study. The authors mentioned the mean SBS values of Quick up (12.6 MPa) to be an acceptable level according to the International Organization for Standardization (ISO) standard. Since the mean SBS value of Quick Up in their study was comparable with this research (11.97 MPa), it can be concluded this material could meet the ISO standard.

In metal surfaces, the airborne particle abrasion alone or followed by the metal primer application has been shown to improve the bond strength of the resins with the metal alloys [5, 12, 14, 15]. The adhesion of PMMA to air abraded CP Ti was over 3.7 times stronger than the untreated one [16]. This method enhances surface energy and the wettability of the metal surface and produces micromechanical roughness [17]. A limitation of this study was the lack of air abrasion treatment of Ti substrate in AMH. Despite the advantages of air abrasion on Ti surface, this treatment was not used in the current study, as this study aimed to find the best chemical adhesion to AMH and an acrylic resin of denture base using primers and adhesives, not through micromechanical retention.

The multimode or universal adhesives such as G-Premio BOND can improve the adhesion of resin materials to substrates, including resin composites, ceramics, zirconia, and metal alloys through functional monomers, such as 10-MDP [18]. The results of this research showed the highest SBS value in Ti surfaces when bonded to a light-cured flowable composite after using G-Premio BOND with and without AP. A variety of metal primers containing different functional groups like 11-methacryloyloxyundecan- 1,1decarboxylic acid (MAC10), 10-methacryloyloxydecyl dihydrogen phosphate (10-MDP), 6-(4-vinylbenzyl-n-propyl)amino-1, 3, 5-triazine-2, 4-dithiol (VBATDT), and methacryloyloxyalkyl thiophosphate derivatives (MEPS) improve the adhesion to Ti substrates [19]. Base on the obtained results of the current study, there was no significant difference between the mean SBS values of GP-P and AP-GP-P and those of Q and AP-Q (*p* =0.63 and *p* =0.65, respectively). Since AP did not positively affect the SBS values, the second hypothesis of this research could not be rejected. AP contains MDP monomer presents great chemical bonding with the Ti oxide layer and copolymerization with the methacrylate-based resin monomer [14], and VBATDT monomer bonds PMMA to noble metals such as gold and palladium [20].

Although most of previous studies confirmed the effectiveness of primers containing 10-MDP monomer on the adhesion of resin materials to Cp Ti or Ti alloys in comparison to carboxylic monomers [15, 17, 19, 21–23], MDP monomer in AP could not improve Ti bonding in this research. It has been determined the conditioning of the Ti surface is important for bonding using MDP-containing monomers. The AP application combined with the airborne particle abrasion with aluminum oxide particles can increase the adhesion to Ti substrate [24]. Indeed, air abrasion increases the micromechanical retention and surface area for bonding and AP promotes wettability and the diffusion of the functional monomers into the irigulaties created by air abrasion thereby decreasing the contact angle for bonding and eventually increases the adhesive bonding of resin materials to Ti. Moreover, the contact of AP with the residual alumina particles resulted in an increase in the bond strength of resins [25]. So, the lack of airborne particle abrasion on Ti surfaces in this study explains the ineffectiveness of AP on bonding to this substrate. In accordance with this finding, a previous study revealed that the application of AP did not improve the bond strength of AMH was retained with Quick up material [1]. The lack of significant improvement in adhesion in the AP-GP-P group may also be attributed to the application of AP before multimode adhesive utilization. Indeed, the presence of 4-MET and phosphoric acid ester monomer in G-Premio BOND, which can improve the bonding to Ti, inhibited the effectiveness of AP. To confirm these statements, a previous study showed that the application of AP before two universal adhesives of Scotchbond Universal Adhesive and G-Premio BOND could not increase the bonding of resin cement to Cp Ti in comparison with using the universal adhesive alone [8]. In agreement, Bushriega et al. [26] have also noted the ineffectiveness of AP in improving the adhesion of the heat-cured acrylic resin to Ti.

Another factor that adversely affected the adhesion of resin materials to Ti following AP utilizing is thermal aging [15, 17, 21, 22]. Kawaguchi et al. [5] showed that after 10,000 cycles of thermal aging the adhesion of AP-treated metals (Co-Cr and CP Ti) to heat-polymerized denture base resin reduced to half of the first bonding value (27.5 ± 4.0 to 14.4 ± 6.8 MPa). Since the whole samples of this research were submitted to thermocycling, the effect of thermal cycles on the adhesion properties of the metal primer could not be investigated.

Based on the obtained results, the application of Monobond Plus as a silane or primer and Multilink Hybrid Abutment cement produced stable and good adhesion to AMH. Silane coupling agents, or briefly silanes, may join essentially different materials together. Since the organofunctional groups of silane can copolymerize with the monomers of resin composites [27], thermal stresses during thermocycling (5–55°C) could not debond the resin samples from Ti substrates. In this connection, Matinlinna et al. [27] reported the highest SBS of Bis-GMA resin to Monobond-S-treated Ti after thermocycling. The pretreatment of Ti surface with Monobond Plus silane and denture base with SR Connect primer before Multilink Hybrid Abutment insertion has been previously recommended for obtaining the favorable adhesion of AMH to resin acrylic of denture base. Regarding the obtained results of this study, this combination was effective on Ti specimens (6.37 ± 2.39 MPa), but it could not produce acceptable adhesion to PMMA samples (0.74 ± 0.45 MPa).

The findings of this study on Ti specimens revealed lower SBS values in Q (2.26 ± 1.98 MPa) and T (4.07 ± 2.37 MPa) groups in which no metal primer and silane were utilized. This finding reiterates the importance of a metal primer containing phosphate monomers such as MDP and silane for attaching resin materials to Ti substrate. It is in line with Yanagida et al. [22] study that using a metal primer before resin bonding to Ti–6Al–7Nb alloy was necessary to withstand thermal stresses during thermocycling.

A factor that may negatively affect the adhesion of resin materials to Ti is the difference in the thermal expansion coefficient of these materials. The thermal expansion coefficient of metal was reported to be approximately one-tenth that of the denture base resin, which facilitated the debonding of metal from the resin surface [27]. Thermocycling accelerates water absorption into the materials and creates a large divergence in the coefficient of thermal expansion between the two bonded materials. Thus, it decreases the bond strength of acrylic resin adhesives to CP Ti and Ti alloys [7, 12, 17, 21, 28]. To prevent separation, the bonding agent must have high adhesive strength and durability to survive internal stresses that will occur at variable temperatures [12]. Although thermocycling was used in this study to simulate the temperature fluctuations of the oral environment in a laboratory setting, the importance of the clinical investigation to confirm the results of this study in the oral environment should not be neglected. Based on ISO 10477 standard [29], 5000 cycles of thermocycling in water baths at temperature range of 5–55 °C is an appropriate regimen for the aging of a metal–resin bond performed in the current research.

This research demonstrated the adhesive failure as the main failure in all study groups. With greater bonding to substrates or the introduction of the chemical adhesion, more cohesive and mixed failures will be expected after debonding. The more cohesive failure in the T group in PMMA samples in this study can be explained by the highest SBS values in this group.

Most overdentures required reattachment of AMH within first few years of clinical service, due to loosening or the complete removal of AMH from the inner surface of the implant-supported overdentures [30]. With the results of this study, it is recommended that clinicians pretreat Ti attachment housings with a multimode universal adhesive (G-Permio BOND) prior to direct transfer with autopolymerizing resin materials (SR Triplex Cold Pink and Quick UP) for obtaining the best bonding strategy to Ti and acrylic resin materials. This study has several limitations that warrant careful consideration before its findings are applied in a clinical setting. The cyclic loading in the oral environment during function may be directly affected the detachment of AMH was not used in this study. Air abrasion and silica coating of Ti activates the substrate surface for covalent chemical bonding through metal primers and silanes and formed the Si─O─Si bonds are hydrolytically stable, so they may improve the adhesion of the resin to Ti surfaces and should be investigated in future studies. The bonding of acrylic resin retaining materials to attachment housing is mainly based on the macromechanical retention through engaging the existing undercuts on the axial walls of the housing. In the literature, the bonding performance of metal components to denture base material was evaluated through the flexural analysis [2, 6] or tensile bond strength, push and pull-out tests [1, 11, 31]. Although a pull-out test design and tensile bond strength measurement might help imitate the intraoral forces that cause debonding of the AMH from the denture base, the SBS test was preferred in this study due to lower technique sensitivity and difficulties in establishing those tests designs. Additionally, when tensile or pull-out forces were applied to the locator housing, they inserted a shear force on the existing acrylic resin around the housing. So, further evaluation using tensile tests are needed for verification. As the longevity of the bond was not also addressed in this in vitro study, further research by applying long-term aging conditions is necessary to clarify the bonding durability. Moreover, longitudinal investigations will be required to reveal clinical performance.

## 5. Conclusions

Despite limitations in this in vitro study, the following conclusions were drawn:

1. In the acrylic resin substrates, utilizing autopolymerizing acrylic resin (SR Triplex Cold Pink) and resin composite (Quick UP) could produce the greatest adhesion which endured the 5000 cycles of thermal aging.
2. The utilization of G-Premio BOND as a universal adhesive with and without prior application of ALLOY PRIMER and the application of Monobond Plus followed by Multilink Hybrid Abutment produced the strongest adhesion to AMH.
3. ALLOY PRIMER could not improve the adhesion of either light-cured or autopolymerized resin materials to the Ti substrate.

## Figures and Tables

**Table 1:** The primer, adhesives, and resin materials were used in this study and their composition details which were provided by the manufacturers.

Materials	Trade name	Composition	Lot number	Manufacturer
Universal or multimode adhesive	G-Premio BOND	Acetone, water, dimethacrylate, 4-MET, phosphoric acid ester monomer, silicone dioxide, photoinitiator pH = 1.5 (moderate)	1609081	GC; Tokyo, Japan

Metal primer	ALLOY PRIMER	Triazine-based vinyl monomer, VBATDT, monomer MDP, solvent (acetone)	6E0085	Kuraray Noritake Dental Inc, Kurashiki, Okayama, Japan

Universal primer agent (silane)	Monobond Plus	Silane methacrylate, Phosphoric acid methacrylate, ethanol and sulfide methacrylate	T29123	Ivoclar Vivadent, Schaan, Liechtenstein

Light curing conditioner	SR Connect	Methyl methacrylate (60%–70%), polymethyl methacrylate (<10%), dimethacrylate (20%–30%) and catalyst (3%–5%)	WL4001	IvoclarVivadent, Schaan, Liechtenstein

Methacrylate-based flowable resin composite	PermaFlo Pink	Bis-GMAB (8.5%), TEGDMAC (20%), 2-Hydroxyethyl methacrylate (HEMA), 2,2,4 Trimethylhexamethylenedicarbonate (TMHEDC), diurethane dimethacrylate, amine methacrylate, organophosphine oxide, sodium monofluorophosphate (0.3%), aluminosilicate glass zirconium filler (68%)	S069	Ultradent, South Jordan, USA

Autopolymerizing luting resin composite	Multilink Hybrid Abutment	Dimethacrylate, HEMA, fillers (barium glass, ytterbium trifluoride, spheroid mixed oxides, titanium dioxide)	U54978	Ivoclar Vivadent, Schaan, Liechtenstein

Autopolymerizing resin acrylic	SR Triplex Cold Pink	Powder polymethyl methacrylate, catalyst, pigments liquid methyl methacrylate stab., dimethacrylate, catalyst, stabilizer	Powder: YT1196 Liquid: V09517	Ivoclar Vivadent, Schaan, Liechtenstein

Heat-polymerized denture base resin	SR Triplex Hot Pink	Powder: polymethyl methacrylate, catalysts (0.5%–1.5% benzoyl peroxide), pigments Liquid: methyl methacrylate stab, dimethacrylate	Powder: N17392 Liquid: N47618	Ivoclar Vivadent, Schaan, Liechtenstein

Autopolymerizing resin composite with bonding agent	Quick Up resin composite	5%–10% 1,6-hexanediylbismethacrylate 1%−2.5% catalyst 2.5% bis-GMA 2.5% benzoyl peroxide	1539091	Voco, GmbH, Germany
	Quick Up bonding agent	50%–100% acetone 2.5%–5% 2-hydroxyethyl methacrylate	1537441	Voco GmbH, Germany

**Table 2:** Mean, standard deviation (SD), minimum (min), and maximum (max) of SBS of bonded specimens to acrylic resin substrates.

Study groups	Number	Mean ± SD	Min	Max	*p* value
P*P*	10	2.52 ± 1.52a^*∗*^	0.78	5.88	*F* = 26.26 *p* < 0.001
A-SR-P	10	8.32 ± 4.51^b,c^	1.46	15.80	
A-SR-Q	10	6.55 ± 3.17^c^	2.27	13.13	
A-SR-MHA	10	0.74 ± 0.45^d^	0.25	1.72	
Q	10	11.97 ± 2.65^b^	8.68	16.19	
T	10	15.11 ± 5.36^b^	6.48	22.50	

Abbreviations: A-SR-MHA, Air-abrasion + SR Connect + Multilink Hybrid Abutment; A-SR-P, Air-abrasion + SR Connect + PermaFlo Pink; A-SR-Q, Air-abrasion + SR Connect + Quick Up; P, Perma Flo Pink; Q, Quick Up; T, SR Triplex Cold Pink.

*⁣* ^*∗*^According to Tukey post hoc test. Different lowercase letters indicate a significant difference among the study groups.

**Table 3:** Mean, standard deviation (SD), minimum (min), and maximum (max) of shear bond strength of bonded specimens to Ti substrates.

Study groups	Number	Mean ± SD	Min	Max	*p* Value
GP-P	10	9.36 ± 1.57a^*∗*^	7.59	12.51	*F* = 9.84 *p* < 0.001
AP-GP-P	10	7.66 ± 3.35^a^	4.03	13.16	
Q	10	2.26 ± 1.98^bd^	0.27	4.85	
AP-Q	10	4.09 ± 2.48^bc^	1.35	8.83	
MP-MHA	10	6.37 ± 2.39^acd^	4.01	11.59	
T	10	4.07 ± 2.37^bcd^	1.90	7.96	

Abbreviations: AP-GP-P, ALLOY PRIMER + G-Premio BOND + PermaFlo Pink; AP-Q, ALLOY PRIMER + Quick Up; GP-P, G-Premio BOND + PermaFlo Pink; MP-MHA, Monobond Plus+ Multilink Hybrid Abutment; Q, Quick Up; T, SR Triplex Cold Pink.

*⁣* ^*∗*^According to Tukey post hoc test, different lowercase letters indicate a significant difference among the study groups.

**Table 4:** The percentage of failure modes in different study groups.

Study groups	Adhesive	Cohesive	Mixed
Acrylic resin groups			
P	100%	0	0
A-SR-P	100%	0	0
A-SR-Q	100%	0	0
A-SR-MHA	100%	0	0
Q	100%	0	0
T	30%	70%	0
AMH groups			
GP-P	100%	0	0
AP-GP-P	100%	0	0
Q	100%	0	0
AP-Q	100%	0	0
MP-MHA	100%	0	0
T	50%	0	50%

## Data Availability

The data used to support the findings of this study are available from the corresponding author upon request.
